# Novel Surgical Approach to Posterior Nasal Neurectomy without Identifying the Posterior Nasal Nerve

**DOI:** 10.1055/s-0045-1808242

**Published:** 2026-04-28

**Authors:** Hirotaka Yamamoto, Tsuyoshi Kojima, Ryusuke Hori

**Affiliations:** 1 Department of Otolaryngology, Tenri Hospital, Nara, Japan; 2 Senbayashiomiya Hara ENT Clinic, Osaka, Japan; 3 Department of Otolaryngology, Head and Neck Surgery, Graduate School of Medicine, Kyoto University, Kyoto, Japan; 4 Department of Otolaryngology-Head and Neck Surgery, School of Medicine, University of Occupational and Environmental Health, Kitakyushu, Japan

**Keywords:** allergic rhinitis, nasal obstruction, sneezing, rhinorrhea, endoscopic posterior nasal neurectomy

## Abstract

**Introduction:**

Posterior nasal neurectomy (PNN), a procedure used to manage intractable allergic rhinitis, requires precise identification of the posterior nasal nerve and associated neurovascular bundles within the posterior nasal region, including the sphenoid artery. Mastery of reliable surgical techniques and considerable experience are imperative for successful execution.

**Objectives:**

We describe a novel surgical strategy for posterior nasal neurectomy that circumvents the need to directly identify the posterior nasal nerve directly.

**Methods:**

To clarify the effects of PNN, pre- and post-operative symptoms, including rhinorrhea, sneezing, and nasal obstruction, were evaluated in patients who underwent PNN using scores derived from the Practical Guidelines for Management of Allergic Rhinitis in Japan 2020. Additionally, preoperative and postoperative (>12 months) medication status and postoperative PNN complications were meticulously documented.

**Results:**

This retrospective study included 80 patients who underwent PNN between January 2016 and December 2021; 46 of the 80 patients agreed to participate in the survey questionnaire, and the responses of these 46 patients were used for the analysis. Postoperatively, all patients were followed up in the outpatient clinic for >12 months (mean, 18 months). Among the 46 patients, the scores for all allergic symptoms showed significant improvement at 12 months postoperatively compared with the preoperative scores. The proportion of patients who received daily anti-allergic medications decreased from 93.5% (43/46) preoperatively to 15.2% (7/46) postoperatively. No severe complications occurred in any patient.

**Conclusions:**

This innovative surgical approach demonstrated efficacy in ameliorating the symptoms associated with allergic rhinitis without concomitantly escalating procedural risks.

## Introduction

Medication is the primary treatment for allergic rhinitis (AR); however, some patients remain unresponsive to conservative therapy. Surgical intervention is recommended in patients with refractory rhinitis. Among the various surgical modalities available for AR, vidian neurotomy is a conventional procedure. Excision of the vidian nerve considerably reduces hypersecretion and hypersensitivity. Nonetheless, vidian neurotomy is accompanied by enduring complications, such as diminished tearing and upper lip numbness. 1 The posterior nasal nerve, a peripheral offshoot of the vidian nerve, originates from the sphenopalatine ganglion anterior to the lacrimal gland. This nerve is the principal conduit for the parasympathetic, sympathetic, and sensory fibers that innervate the nasal respiratory mucosa. Posterior nasal neurectomy (PNN) is a novel alternative technique in which the neurovascular bundle is selectively severed to circumvent the surgical ramifications of vidian neurotomy, particularly reduced lacrimation. 2 Consequently, in recent decades, PNNs have witnessed an upsurge in their application for the management of recalcitrant severe AR. In the conventional approach to the PNN, the neurovascular bundle comprising the posterior nasal nerve and sphenopalatine artery at the sphenopalatine foramen is identified and sectioned following the meticulous elevation of the mucoperiosteal flap. 3 4

Moreover, submucosal inferior turbinectomy (SIT) is more effective in alleviating intractable nasal congestion stemming from inferior turbinate hypertrophy, demonstrating superior effectiveness. 5 Therefore, PNN combined with the SIT is frequently performed in patients with severe AR and inferior turbinate hypertrophy. 6 Proficiency in surgical techniques and substantial experience are requisite for PNN to navigate the dissection of structures around the sphenopalatine foramen, with primary concerns centered on intraoperative and/or postoperative bleeding. In the present study, we devised a unique procedure for the PNN that obviates the need to identify the posterior nasal nerve. The peripheral branches of the posterior nasal nerve were concurrently severed through a mucoperiosteal incision along the base of the inferior turbinate without raising the mucoperiosteal flap or directly pinpointing the posterior nasal nerve around the sphenopalatine foramen. We aimed to assess the efficacy of this unique procedure for managing severe AR in PNN with or without SIT.

## Methods

### Study Design and Participants

A retrospective analysis was conducted on patients who underwent PNN between January 2016 and December 2021 for persistent symptoms of perennial allergic rhinitis despite receiving medication for >3 months. The local Ethics Committee of the Hospital approved the study protocol. Subjective symptoms including rhinorrhea, sneezing, and nasal obstruction were evaluated using a questionnaire based on the Practical Guidelines for the Management of Allergic Rhinitis in Japan 2020. Each symptom was assessed on a scale of 0–4 (0 = none, 1 = mild, 2 = moderate, 3 = severe, and 4 = very severe). 7 The patients completed the questionnaire both preoperatively and 12 months postoperatively. The preoperative and 3-month postoperative medication use statuses and postoperative complications from the first day after surgery to the end of the follow-up period were documented. Numerical data are presented as the arithmetic mean ± standard deviation, while categorical variables are presented as relative frequencies (n) and percentages. Repeated measures analysis of variance was used to examine the effect over time (at multiple points in time), whereas the paired t-test was used to assess the statistical difference between two points. The exclusion criteria included other conditions associated with rhinorrhea and nasal obstruction, such as nasal polyposis, chronic sinusitis, idiopathic (vasomotor) rhinitis, non-allergic rhinitis with eosinophilia syndrome, and autoimmune diseases. Patients with incomplete preoperative clinical evaluations, those who missed postoperative outpatient recheck-ups, or those who declined to participate were excluded.

### Surgical Procedure

**Video 1** A new simplified method for endoscopic posterior nasal neurectomy in the management of allergic rhinitis.


**Fig. 1:**
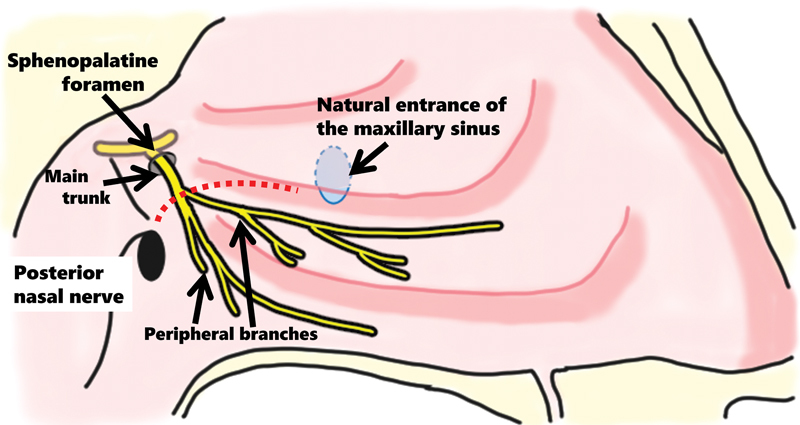
The incision line along the base of the inferior nasal turbinate. An incision line is depicted along the base of the inferior nasal turbinate, extending from its posterior end to the maxillary sinus ostium (represented by a broken line). The peripheral branches of the posterior nasal nerve can be simultaneously divided.

**Fig. 2:**
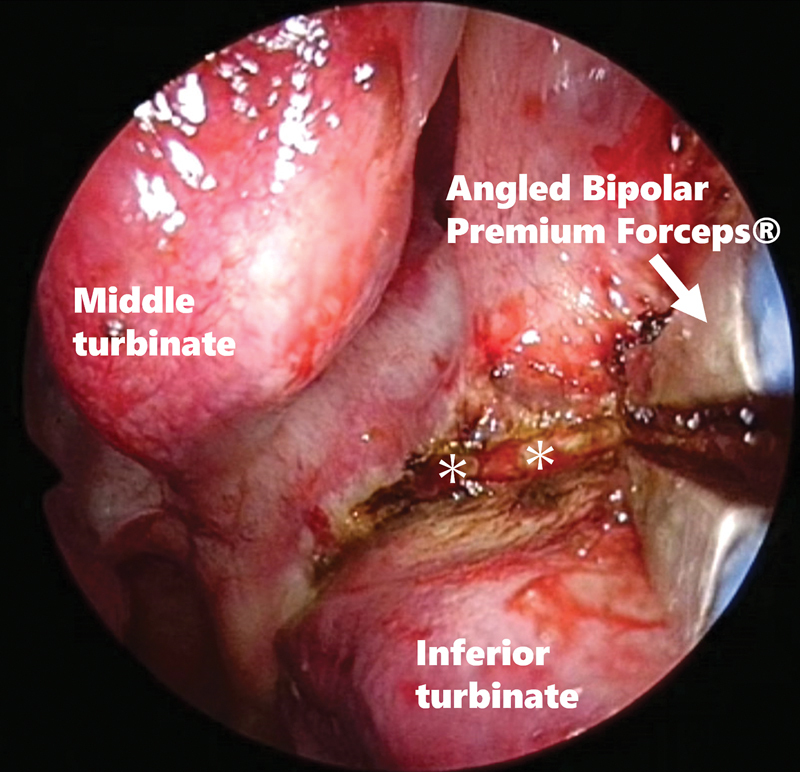
Mucoperiosteal incision up to the bony turbinate using Angled Bipolar Premium Forceps®. Asterisk: the inferior turbinate bone exposed through a mucoperiosteal incision.

The patient was placed in the supine position and underwent surgery under general or local anesthesia. All surgical interventions (Supporting Video 1 ) were conducted using a 4 mm 0° nasal endoscope. Figure 1 illustrates the incision line for the PNN procedure used in this study. Along the inferior nasal turbinate base, an incision was made at the posterior end adjacent to the maxillary sinus ostium ( Fig. 1 ). After adequate coagulation of the mucosal incision line, a full thickness mucoperiosteal incision was made up to the bony turbinate using energy devices, including angled bipolar electrocauteries (bayonet type Bipolar Premium Forceps ^®^ , VIO ^®^ 3, ERBE; Germany). Bipolar Premium Forceps ^®^ with soft coagulation and auto-cut modes facilitated incision post-sufficient coagulation of the mucoperiosteum ( Fig. 2 ). In addition, these energy devices mitigate the risks of intraoperative and postoperative hemorrhages. The posterior nasal nerve was not directly visualized because no mucoperiosteal flap was elevated, and the mucoperiosteum was incised along the base of the inferior nasal turbinate. Subsequently, the peripheral branches of the posterior nasal nerve were simultaneously severed.

**Fig. 3:**
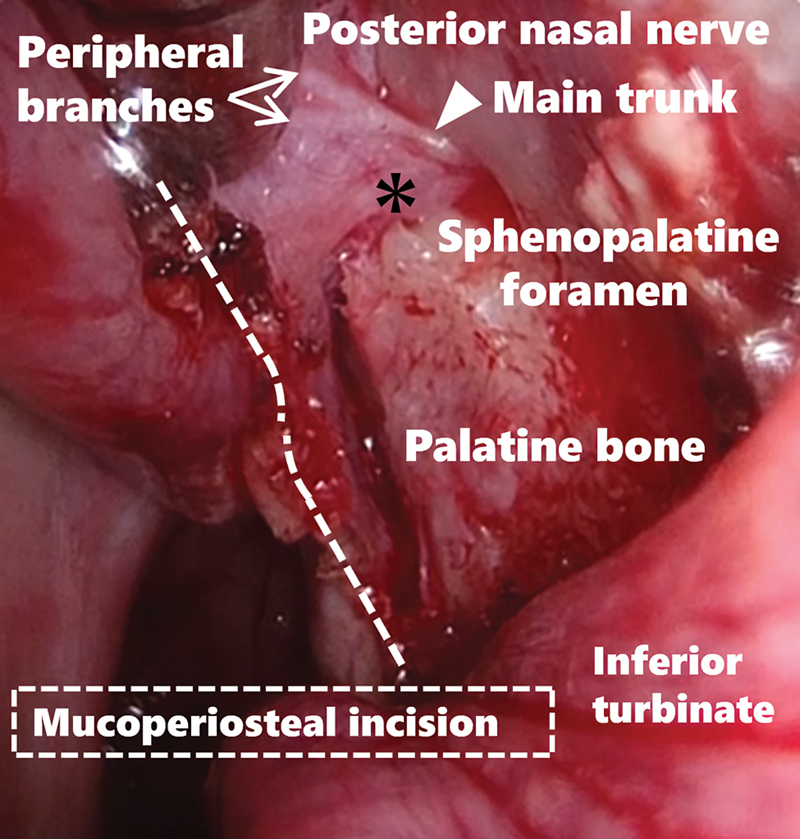
Intraoperative findings after the elevation of the mucoperiosteal flap. The mucoperiosteal flap is elevated posteriorly from the incision line at the posterior end of the inferior nasal turbinate. The neurovascular bundle containing the peripheral branches of the posterior nasal nerve and the sphenopalatine artery was divided. Arrow: peripheral branches of the posterior nasal nerve, Arrowhead: main trunk of the posterior nasal nerve, Asterisk: sphenopalatine artery.

Although not typically employed in this surgical approach, if a mucoperiosteal flap is elevated posteriorly from the mucosal incision line at the posterior end of the inferior nasal turbinate, the neurovascular bundle housing the posterior nasal nerve and peripheral branches of the sphenopalatine artery is severed ( Fig. 3 ).

If SIT reduction was required for nasal obstruction caused by inferior turbinate hypertrophy, SIT was performed using the procedure reported by House et al. 8 They suggested the efficacy of the anterior one-third of the turbinate bone, even in patients with mucosal hypertrophy, in minimizing interference with the physiological function of the inferior turbinate mucosa. A mucosal incision was made over the anterior-inferior aspect of the turbinal bone, followed by elevation of the mucoperiosteum 1.5 cm from the anterior margin and excision of the anterior one-third of the turbinal bone to reduce the volume of the inferior nasal turbinate. After endonasal surgery, the middle nasal meatus was filled with a small amount of calcium alginate fiber (Algoderm ^TM^ ) to enhance hemostasis and facilitate wound healing at the surgical sites.

### Statistical Analysis

Numerical data are presented as the arithmetic mean ± standard deviation, whereas categorical variables are presented as relative frequencies (n) and percentages. Repeated-measures analysis of variance was used to examine the effect over time (at different multiple points in time), whereas the paired t-test was used to examine the statistical difference between two points in time. Statistical significance was defined as p < 0.05. All statistical analyses were performed using EZR (Saitama Medical Center, Jichi Medical University), a graphical user interface for R (The R Foundation for Statistical Computing, Vienna, Austria, version 2.13.0). 9

## Results

Eighty patients with intractable AR underwent the PNN procedure during registration and 46 completed the survey. Among the respondents, 29 were males (63.0%) and 17 were females (37.0%), with ages ranging from 15–79 years (mean age, 45.91 ±  20.59 years). Forty-two patients underwent the PNN procedure in conjunction with the SIT, whereas four underwent the PNN procedure alone. All patients underwent postoperative follow-up evaluations at the outpatient clinic for more than 12 months (mean follow-up duration, 18 months).

**Fig. 4:**
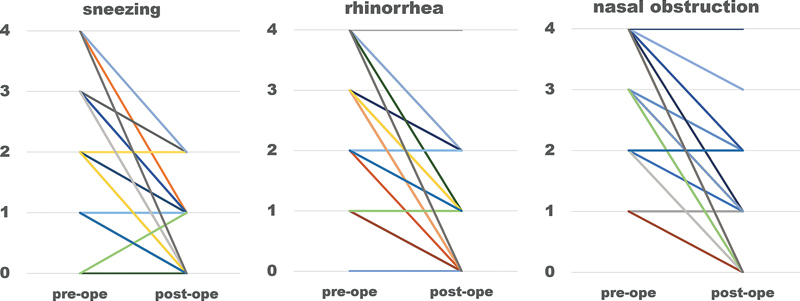
Preoperative and postoperative nasal-symptom evaluation scores. Statistical significance is denoted by *p < 0.001.

Regarding clinical efficacy, significant improvements in symptom scores were observed at 12 months postoperatively compared to the preoperative scores. Scores for rhinorrhea decreased from 2.76 ± 1.20 to 1.00 ± 0.84 (p <0.01), sneezing decreased from 2.02 ± 1.16 to 0.67 ± 0.67 (p <0.01), and nasal obstruction decreased from 3.26 ± 0.83 to 1.07 ± 0.90 (p <0.01) ( Fig. 4 ). The four patients who underwent only PNN procedures also showed improvement: rhinorrhea decreased from 4 to 0, sneezing decreased from 3.5 to 0, and nasal obstruction decreased from 4 to 0. The percentage of patients requiring daily anti-allergic medication decreased from 93.5% (43/46) preoperatively to 15.2% (7/46) postoperatively. Furthermore, 54.3% (25/46) of the patients were able to discontinue medications postoperatively; however, two patients experienced symptom relapse at 7 and 13 months postoperatively, necessitating occasional medication.

No severe complications were reported among the patients; however, one patient experienced postoperative nasal hemorrhage 21 days post-surgery, which was effectively managed with nasal packing. The patient had minor crusting observed for 2 weeks postoperatively, which might have prolapsed, resulting in a hemorrhage.

## Discussion

The posterior nasal nerve contains parasympathetic and afferent sensory nerve fibers within the nasal respiratory mucosa. The PNN effectively alleviates and manages nasal secretion and sneezing by severing parasympathetic and afferent sensory nerve fibers. Sonoda et al. introduced an endoscopic surgical approach to identify and divide the posterior nasal nerve in the sphenopalatine foramen by raising a mucoperiosteal flap after septoplasty and inferior turbinate reduction. The authors reported satisfactory outcomes in patients with refractory AR. 6 In the present study, the posterior nasal nerve was not identified under direct view, and the peripheral branches of the posterior nasal nerve were divided at the base of the inferior turbinate without elevating a mucoperiosteal flap or exposing the main trunk of the posterior nasal nerve. This unique procedure for PNN offers several advantages. It is simple and less invasive; therefore, it can be performed under local anesthesia.

Furthermore, intra-and postoperative hemorrhages rarely occur because the artery is sufficiently coagulated and divided distally from the sphenopalatine foramen. However, most reports suggest identification and division of the posterior nasal nerve near the sphenopalatine foramen. This is because the posterior nasal nerve divides into several branches after passing through the sphenopalatine foramen, and surgeons may miss other branches if they target peripheral branches. However, in the present study, the peripheral branches divided immediately after arising from the main trunk of the posterior nasal nerve ( Fig. 3 ). Additionally, the results of this study are comparable to those of previous reports. Therefore, this surgical procedure for PNN should be considered an effective treatment for sneezing and rhinorrhea associated with AR.

Among the rhinological surgeries to alleviate nasal symptoms, PNN has been shown to ease sneezing and rhinorrhea. 2 10 In contrast, SIT reduction reduces the volume of the inferior turbinate, thereby mitigating nasal obstruction. Consequently, a combination of PNN and SIT has been frequently used to manage refractory severe AR accompanied by inferior turbinate hypertrophy. In this study, nearly 90% of the participants underwent both the PNN and SIT, which significantly enhanced clinical scores related to symptoms such as rhinorrhea, sneezing, and nasal obstruction. Garcia et al. reported that SIT alone did not alleviate the symptoms of nonobstructive allergic rhinitis in the inferior nasal turbinate. 11 Moreover, our SIT procedure only addresses the anterior one-third of the inferior turbinate even if nasal obstruction is improved; therefore, it has limited efficacy in reducing rhinorrhea and sneezing in this region. The SIT did not affect the posterior part of the inferior turbinate because of the anatomical distribution of the posterior nasal nerve. However, the extent to which the effect of SIT exceeds that of PNN on rhinorrhea and sneezing in patients treated with both surgeries is unknown, which is a limitation of this study. On the other hand, the peripheral branches of the posterior nasal nerve were cut, as shown in Figure 3 . Additionally, all nasal symptoms improved among patients who exclusively underwent PNN, which is potentially attributable to the secondary effects of reduced nasal secretion. 5 These findings suggest that the effect of the PNN observed in this investigation is comparable to that reported for conventional PNN techniques.

This retrospective study has several limitations. First, because of the innervation of the posterior half of the middle turbinate by the posterior nasal nerve and the presence of numerous submucosal glands within the middle turbinate, 12 the PNN procedure implemented in this study could not address hypersecretions from the posterior segment of the middle turbinate. Nevertheless, given the relatively smaller surface area of the middle turbinate compared to the inferior turbinate, the outcomes of this study demonstrated the amelioration of nasal symptoms following the PNN procedure. Consequently, further exploration of the posterior nasal branch of the middle turbinate may be unnecessary. Second, all symptom scores improved with this method; however, these scores are subjective and objective assessments of nasal conditions, such as rhinomanometry and histological examination of the nasal mucosa. Third, this study was short, involved a limited number of cases, and required a control group. Subsequent large-scale investigations with extended follow-up periods are necessary to comprehensively assess the effects.

## Conclusion

Here, we present a novel surgical technique for posterior nasal neurectomy that does not involve the direct identification of the posterior nasal nerve. This distinctive approach offers the benefits of simplicity and reduced invasiveness independent of the surgeon's skill level. No significant peri-operative complications were observed. Posterior nasal neurectomy described in this study is a valuable surgical intervention.
